# Supplementary material for: Differential ex vivo susceptibility of Plasmodium malariae and Plasmodium falciparum clinical isolates from Ghana and Mali to current and lead discovery candidate antimalarial drugs
Source: Microbiol Spectr. 2025 Mar 10;13(4):e02176-24. doi: 10.1128/spectrum.02176-24 (PMC11960124; doi:10.1128/spectrum.02176-24)
Supplement: Supplemental tables — Tables S1 to S3. [file spectrum.02176-24-s0001.docx]

Supplementary Tables

Supplementary Table 1: Fifty percent inhibitory concentration (IC_50_) of reference approved antimalarial against P. malariae and P. falciparum in Ghana.

|  | IC_50_ (nM) of reference antimalarial drugs against *P. malariae* and *P. falciparum* clinical field isolates, Ghana | | | | | | | | | | | | | | | | |
| --- | --- | --- | --- | --- | --- | --- | --- | --- | --- | --- | --- | --- | --- | --- | --- | --- | --- |
|  | *P. malariae* field isolates | | | | | | | |  | *P. falciparum* field isolates | | | | | | | |
|  | CQ | QN | ART | Pyr | SFX | LUM | AVQ | TFN |  | CQ | QN | ART | Pyr | SFX | LUM | AVQ | TFN |
| Number of isolates | 11 | 11 | 11 | 11 | 11 | 11 | 11 | 11 |  | 10 | 10 | 10 | 10 | 10 | 10 | 10 | 10 |
| Median IC_50_ (nM) | 30.32 | 19.25 | 7.12 | 8.73 | 25.72 | 39.46 | 10.38 | 3.13 |  | 18.32 | 15.35 | 2.15 | 14.86 | 7.86 | 23.06 | 2.51 | 3.63 |
| Mean IC_50_ (nM) | 40.84 | 20.16 | 9.06 | 10.72 | 33.40 | 53.32 | 11.44 | 5.04 |  | 26.41 | 20.87 | 3.12 | 39.30 | 9.77 | 35.50 | 3.48 | 4.84 |
| Range CI95 (nM) | 9.441 - 115.7 | 13.43 - 30.12 | 1.33 - 29.15 | 4.395 - 25.19 | 14.33 - 100.1 | 5.154 - 160.7 | 5.183 -22.32 | 1.028 -11.16 |  | 10.36 - 69.31 | 9.265 - 5.33 | 0.9760 - 9.135 | 1.356 - 214.7 | 1.379 - 25.13 | 2.365 - 134.3 | 0.5790 - 9.326 | 0.9640 - 11.33 |

^Footnote: CQ= Chloroquine, QN= Quinine, ART= Artemether. Pyr= Pyrimethamine, SFX= Sulfadoxine, LUM= Lumefantrine, AVQ= Atovaquone, TFN= Tafenoquine, PPQ= Piperaquine^

Supplementary Table 2: Fifty percent inhibitory concentration (IC_50_) of reference approved antimalarial against P. malariae and P. falciparum in Mali.

|  | IC_50_ (nM) of reference antimalarial drugs against *P. malariae* and *P. falciparum* clinical field isolates, Mali | | | | | | | | | | | | | | | | | | |
| --- | --- | --- | --- | --- | --- | --- | --- | --- | --- | --- | --- | --- | --- | --- | --- | --- | --- | --- | --- |
|  | *P. malariae* field isolates | | | | | | | | |  | *P. falciparum* field isolates | | | | | | | | |
|  | CQ | QN | ART | Pyr | SFX | LUM | AVQ | TFN | PPQ |  | CQ | QN | ART | Pyr | SFX | LUM | AVQ | TFN | PPQ |
| Number of isolates | 16 | 16 | 16 | 16 | 16 | 16 | 16 | 16 | 13 |  | 12 | 11 | 11 | 11 | 12 | 11 | 11 | 11 | 11 |
| Median IC_50_ (nM) | 17.83 | 18.35 | 2.55 | 9.15 | 6.62 | 18.34 | 4.01 | 5.55 | 27.63 |  | 24.05 | 26.84 | 2.05 | 7.84 | 21.5 | 18.46 | 3.82 | 2.85 | 26.94 |
| Mean IC_50_ (nM) | 132.5 | 20.06 | 47.24 | 8.72 | 6.63 | 50.51 | 4.16 | 6.37 | 27.55 |  | 115.5 | 27.75 | 2.52 | 9.88 | 20.46 | 23.07 | 3.92 | 3.25 | 27.57 |
| Range (CI95) | 8.48 -718.6 | 11.73 - 34.64 | 0.83 - 713.7 | 3.92 - 13.48 | 3.94 - 9.24 | 9.27 - 521.5 | 1.38 - 9.33 | 3.75 - 11.53 | 17.73 - 42.74 |  | 14.63 - 1033 | 16.73 - 47.71 | 0.63 - 4.71 | 5.84 - 21.94 | 6.84 - 36.81 | 13.64 - 41.64 | 2.11 - 6.63 | 1.63 - 6.84 | 15.63 - 37.72 |

Supplementary Table 3: Fifty percent inhibitory concentration (IC_50_) of antimalarial drug candidates against P. malariae and P. falciparum in Mali.

|  |  | IC_50_ (nM) against *P. malariae* field isolates *P. falciparum* clinical field isolates, Mali | | | | | | | |
| --- | --- | --- | --- | --- | --- | --- | --- | --- | --- |
|  |  | | | |  |  | | | |
|  | INE963 | MMV1579167 | MMV1581373 | MMV1793609 |  | INE963 | MMV1579167 | MMV1581373 | MMV1793609 |
| Number of isolates | 12 | 12 | 12 | 12 |  | 12 | 11 | 11 | 11 |
| Median IC_50_ (nM) | 1.86 | 2.89 | 6.28 | 2.18 |  | 2.50 | 20.45 | 11.85 | 16.24 |
| Mean IC_50_ (nM) | 2.38 | 2.99 | 5.75 | 3.12 |  | 2.41 | 20.50 | 13.28 | 18.08 |
| Range (CI95) | 8.893- 6.382 | 0.9940- 5.227 | 0.491- 11.36 | 1.006 - 6.295 |  | 1.035-4.001 | 9.503 - 34.07 | 3.215 - 31.48 | 7.038 - 43.96 |
